# Baseline lung function and risk of incident tuberculosis: a nationwide population-based cohort study

**DOI:** 10.1371/journal.pone.0322616

**Published:** 2025-04-29

**Authors:** Kyu Yean Kim, Yong-Moon Park, Seung Hyun Ko, Kyungdo Han, Seung Hoon Kim, Shin Young Kim, Sung Kyoung Kim

**Affiliations:** 1 Division of Pulmonology and Critical Care Medicine, Department of Internal Medicine, Uijeongbu St. Mary’s Hospital, College of Medicine, The Catholic University of Korea, Seoul, Republic of Korea; 2 Department of Epidemiology, Fay W. Boozman College of Public Health, University of Arkansas for Medical Sciences, Little Rock, Arkansas, United States of America; 3 Division of Endocrinology and Metabolism, Department of Internal Medicine, St. Vincent’s Hospital, College of Medicine, The Catholic University of Korea, Suwon, Republic of Korea; 4 Department of Statistics and Actuarial Science, Soongsil University, Seoul, Republic of Korea; 5 Division of Pulmonary and Critical Care Medicine, Department of Internal Medicine, St. Vincent’s Hospital, College of Medicine, The Catholic University of Korea, Seoul, Republic of Korea; University of Cape Town, SOUTH AFRICA

## Abstract

**Background:**

While lung function is known to decrease after suffering from pulmonary tuberculosis (TB), little is known about whether baseline lung function is associated with the occurrence of TB in the general population. This study aimed to evaluate the risk of incident TB according to baseline lung function.

**Methods:**

A nationwide population-based cohort study was conducted using a database derived by cross-referencing Korea National Health and Nutrition Examination Survey and National Health Insurance Service between 2010 and 2016. Of 29,524 subjects, 16,594 participants aged over 40 years who had spirometry results without a previous TB history were enrolled. The primary endpoint was newly developed TB.

**Results:**

Among 16,457 participants, 72 were newly diagnosed with TB during the follow-up period (median: 5.5 years). TB risk was higher in participants with obstructive lung function impairment (aHR: 2.033, 95% CI: 1.123–3.679) or restrictive lung function impairment (aHR: 2.193, 95% CI: 1.120–4.294) than in those with normal lung function. Low forced expiratory volume in one second (FEV1) was associated with increased TB risk (aHR [lowest quartile vs. highest quartile]: 1.91, 95% CI: 1.05–3.50; aHR [lowest decile vs. highest decile]: 2.76, 95% CI: 1.14–6.70; both *p* for trends < 0.0001).

**Conclusion:**

Our findings suggest that impaired lung function might increase TB risk and that TB risk might be inversely associated with FEV_1_.

## Introduction

According to Global Tuberculosis Report 2020, Tuberculosis (TB) is the leading infectious disease and one of the top 10 causes of death overall around the world. In South Korea, incidence and mortality rates of TB are 77 and 5.2 per 100,000 people, respectively, which are the highest among members of the Organization for Economic Cooperation and Development (OECD) [1]. Because TB has been a major public health threat in South Korea, the government has tried to control TB by providing support multidimensionally [2].

Most previous studies have reported that pulmonary function is decreased after pulmonary TB infection [3,4]. Jung *et al*. have analyzed 822 subjects with a history of pulmonary TB among approximately 15,000 subjects based on National Health and Nutrition Examination Survey [5]. They found that 29.1% of those who had received pulmonary TB treatment in the past had airflow obstruction. This means that about 1 in 3 people who have had a pulmonary TB in the past will develop chronic obstructive pulmonary disease (COPD). They also showed that the risk of developing COPD in those who had a history of pulmonary TB was 2.3 times higher than in those without such history [5]. Another study has reported that a history of pulmonary TB is associated with spirometric restriction in addition to obstructive airflow limitation [6].

However, little is known about the relationship between baseline lung function and the occurrence of TB in the general population. Thus, the aim of the present study was to determine whether baseline lung function had an association with TB development among the general population using a large, nationwide database.

## Materials and methods

### Source of the database and study population

Data used for this study were obtained by cross-referencing the Korea National Health and Nutrition Examination Survey (KNHANES) and the National Health Insurance Service (NHIS) data. Conducted by the Korean Centers for Disease Control and Prevention since 1998, KNHANES is a regular survey that assesses the health and nutritional status of the non-institutionalized civilian population in Korea [7]. NHIS is a social insurance payment system encompassing approximately 97% of the Korean population. The NHIS dataset encompasses comprehensive health check-up data, claims data, prescription information, diagnostic codes using the International Classification of Disease-10 (ICD-10) system, and details of claimed treatments [8].

**Fig 1:**
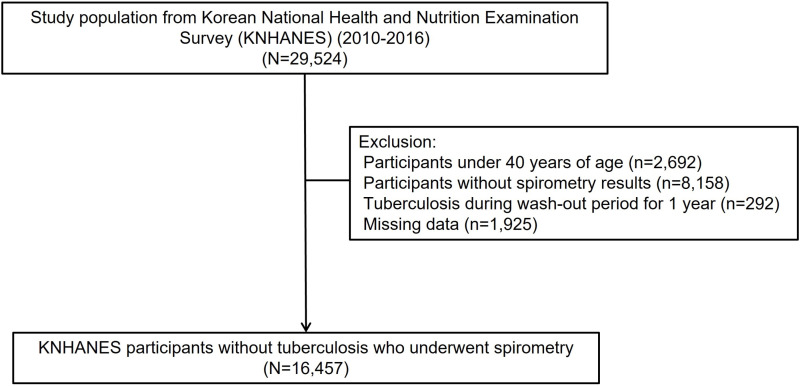
Flow chart of the study population. 16,457 participants were enrolled in this study.

In this research, we utilized national data from the KNHANES collected between 2010 and 2016. Of 29,524 NHANES participants, our analysis focused on adults aged 40 years or more who had undergone spirometry (Fig 1). We excluded individuals with missing values and those who had already been diagnosed with TB prior to January 1, 2010. A washout period exceeding 1 year was established to ensure identification of newly diagnosed TB cases. To assess newly diagnosed TB, we employed data obtained from a 2010 NHIS cohort, with clinical follow-up for the primary outcome extending until December 31, 2018.

### Ethical considerations

The protocol of this study was exempted from the Institutional Review Board (IRB) of Catholic University of Korea (IRB no: UC21ZASI0079), because this study was conducted using publicly available data from 2010 to 2018 provided by the KNHANES and NHIS in the Republic of Korea. The NHIS approved the provision of suitable data, which contained no identifiable personal information, for this study. Since the data could only be accessed at the NHIS Analysis Center, the researcher made periodic visits to this facility to conduct data analysis from September 2020 to August 2021, following the completion of data construction for the study.

### Clinical and laboratory measurements

Details of KNHANES regarding health surveys, standardized physical examinations, laboratory tests, and definition of risk factors have been described in a previous paper [8]. Physical examinations were conducted by specially trained examiners using standardized techniques. Body mass index (BMI) was calculated as the participant’s weight in kilograms divided by the square of their height in meters. Waist circumference was measured at the midpoint between the lowest rib and the anterior iliac crest while standing. Health-related behavior surveys employed validated questionnaires to gather demographic and socioeconomic information. Smoking status was categorized as nonsmoker, ex-smoker, or current smoker. Alcohol consumption was evaluated based on the average number of drinks and frequency of consumption. Heavy drinkers were identified as those consuming over 30 g/day, while those consuming less than 30 g/day were classified as mild to moderate drinkers [9]. Moderate physical activity was defined as walking for at least 150 minutes per week [9]. Household income was divided into quartiles: lowest, lower middle, higher middle, and highest. A high education level was defined as completing high school education or higher.

Diabetes mellitus (DM) was identified by a fasting glucose level ≥ 126 mg/dL, current use of anti-diabetic medications, or a self-reported physician diagnosis of DM [10]. Hypertension was characterized by systolic blood pressure ≥ 140 mmHg or diastolic blood pressure ≥ 90 mmHg, current use of anti-hypertensive medications, or a self-reported physician diagnosis of hypertension [11]. Hypercholesterolemia was determined by total cholesterol level ≥ 240 mg/dL, the use of cholesterol-lowering medications, or a self-reported physician diagnosis of hypercholesterolemia [11]. Blood samples were obtained following an overnight fast.

### Spirometry

Spirometry was performed by four technicians, each of whom had undergone two education sessions on lung function test and quality control. Trained technicians measured forced expiratory volume in 1 second (FEV1), forced vital capacity (FVC), and the FEV_1_/FVC ratio using a dry rolling seal spirometer (model 2130; Sensor Medics, Yorba Linda, CA, USA) in accordance with the American Thoracic Society/European Respiratory Society criteria for the standardization of lung function test [12]. All spirometry values were reported as prebronchodilator results [13]. Normal predictive values were established based on age, sex, height, and ethnicity from large population studies of healthy individuals [14]. Data analysis was limited to results meeting the following criteria: (i) two acceptable spirometry curves displaying correct test initiation and expiration for a minimum of 6 seconds, and (ii) a maximum difference of < 150 mL between two FEV1 or FVC measurements. Spirometry outcomes were categorized into three groups: normal, restrictive, and obstructive lung function. Participants with an FEV1/FVC ratio ≥ 0.7 and FVC ≥ 80% of the normal predicted value were deemed normal. A restrictive pattern was defined as those with an FEV1/FVC ratio ≥ 0.7 and FVC < 80% the normal predicted value, while an obstructive pattern was identified by an FEV1/FVC ratio < 0.7 [15].

### Clinical outcomes

The primary outcome was the identification of newly diagnosed TB cases during the follow-up period. Since 2005, the Korean government has introduced a policy to expand NHIS benefit coverage, offering financial protection against TB to reduce its national burden. This NHIS program covers 100% of TB treatment costs. Upon registering patients with TB in this system, they are assigned a specific code (V code: V206 or V246). We identified TB patients using both ICD-10 codes (A15-A19) and V codes following the approach used in a prior study [16].

### Statistical analysis

Summary statistics are presented as means and standard deviations for continuous variables or as counts and percentages for categorical variables. Continuous variables were compared using Student’s t-test or analysis of variance as appropriate, while categorical variables were compared using the chi-square test. The incidence rate of TB was determined by dividing the number of incident TB cases by the total number of person-years of follow-up and expressed as per 1000 person-years. Participants were followed until the first TB diagnosis, censoring due to death, or December 31, 2016. Cumulative incidence function of TB was produced using the Kaplan-Meier estimate and compared with the log-rank test. The association between lung function and TB risk was analyzed using Cox proportional hazards models, with hazard ratio (HR) and 95% confidence interval (CI) calculated. Multivariable regression models were built using a non-adjustment model (model 1), a model after adjusting for age, sex, BMI, smoking, alcohol consumption, household income, and exercise (model 2), and a model with further adjustment for DM, hypertension, and hypercholesterolemia in addition to model 2 variables (model 3). All statistical analyses were conducted using SAS version 9.4 (SAS Institute, Cary, NC, USA). All *P*-values provided are two-sided, with the level of significance set at 0.05.

## Results

### Study population

There were 29,524 participants derived from KNHANES and NHIS between 2010 and 2016. In the KNHANES, the target age for spirometry is 40 years of older. Therefore, subjects under 40 years of age were excluded from the study. In addition, Subjects who did not undergo spirometry for any reason were also excluded from the study. Those who were diagnosed with TB during wash-out period for 1 year were also excluded. A flow chart of participant selection is shown in Fig 1. Participants (n = 16,457) were divided into three groups based on spirometry as follows: normal, 12,666 (77.0%); obstructive pattern, 2,204 (13.4%); and restrictive pattern, 1,587 (9.6%) (Table 1). Proportions of aged person older than 65 years, male, smokers, heavy alcoholics, less educated, lowest quartile of household income, DM, and hypertension were significantly higher in participants with obstructive or restrictive lung function impairment than in participants with normal lung function. Subjects with restrictive lung function impairment had higher mean BMI and waist circumference than subjects with obstructive lung function impairment or normal lung function. They were followed up until December 31, 2018.Among a total 16,457 participants, 72 were newly diagnosed with TB during the follow-up period. Median follow-up period was 5.5 years (interquartile range: 3.5–7.5 years). Baseline clinical characteristics of participants with or without TB are shown in Table 2. There was no statistically significant difference in sex, smoking status, or alcohol consumption between participants with TB and those without TB. The mean age of participants with TB was significantly higher than that of participants without TB (65.24 ± 10.7 years versus 57.95 ± 10.51 years, *p *< 0.0001). The proportion of participants older than 65 years was significantly higher in participants with TB (61.11% versus 28.64%, *p *< 0.0001). Regarding socioeconomic status (SES), the percentage of participants with high education level was lower in participants with TB than in those without TB (38.89% versus 57.7%, *p *= 0.0013). Also, the percentage of lowest quartile of household income was higher in participants with TB (31.94% versus 18.6%, *p *= 0.0037). The prevalence of DM and hypercholesterolemia was significantly higher in participants with TB than in those without TB.

**Table 1:** Baseline characteristics of participants according to lung function.

	Normal	Obstructive pattern	Restrictive pattern	*p*-value
Parameters	12,666 (77.0%)	2,204 (13.4%)	1,587 (9.6%)	
Age	56.19 ± 9.97	65.97 ± 9.36	61.18 ± 10.35	<.0001
Age ≥ 65 year	2769 (21.86%)	1327(60.21)	641 (40.39%)	<.0001
Sex				<.0001
Male	4870 (38.45%)	1628 (73.87%)	759 (47.83%)	
Female	7796 (61.55%)	576 (26.13%)	828 (52.17%)	
Smoking				<.0001
Non	8347 (65.9%)	705 (31.99%)	938 (59.11%)	
Ex	2329 (18.39%)	874 (39.66%)	369 (23.25%)	
Current	1990 (15.71%)	625 (28.36%)	280 (17.64%)	
Alcohol consumption				<.0001
Non	3723 (29.39%)	697 (31.62%)	539 (33.96%)	
Mild	8028 (63.38%)	1275 (57.85%)	920 (57.97%)	
Heavy	915 (7.22%)	232 (10.53%)	128 (8.07%)	
Education ≥ high school	7699 (60.78%)	972 (44.1%)	811 (51.1%)	<.0001
Household Income (Q1)	2029 (16.02%)	671 (30.44%)	371 (23.38%)	<.0001
Physical Activity (walk)	4738 (37.41%)	903 (40.97%)	541 (34.09%)	<.0001
Diabetes Mellitus	1391 (10.98%)	426 (19.33%)	397 (25.02%)	<.0001
Hypertension	4467 (35.27%)	1103 (50.05%)	849 (53.5%)	<.0001
Hypercholesterolemia	2669 (21.07%)	456 (20.69%)	429 (27.03%)	<.0001
BMI	24.13 ± 2.88	23.78 ± 2.74	25.37 ± 3.24	<.0001
Waist circumference	77.41 ± 9.02	80.01 ± 8.81	81.99 ± 9.47	<.0001
Glucose	100.42 ± 22.01	104 ± 22.5	109.19 ± 30.45	<.0001
SBP	120.95 ± 16.39	125.38 ± 16.51	126.64 ± 16.71	<.0001
DBP	77.45 ± 10.05	75.61 ± 10.35	77.49 ± 10.44	<.0001
Total Cholesterol	196.24 ± 36.01	188.42 ± 35.93	193.2 ± 38.11	<.0001
HDL-C	50.15 ± 12.07	47.39 ± 11.97	47.16 ± 11.74	<.0001
FEV_1_	96.46 ± 10.28	79.23 ± 14.89	77.89 ± 7.78	<.0001
FVC	95.51 ± 9.38	90.78 ± 13.68	74.26 ± 5.53	<.0001
FEV_1_/FVC	0.8 ± 0.05	0.64 ± 0.06	0.79 ± 0.05	<.0001
AST*	21.55 (21.43-21.67)	22.82 (22.52-23.12)	23.36 (22.97-23.75)	<.0001
ALT*	18.97 (18.81-19.13)	19.41 (19.05-19.79)	21.87 (21.33-22.43)	<.0001
TG*	118.21 (117.03-119.41)	125.47 (122.65-128.35)	133.6 (129.94-137.36)	<.0001

**Table 2:** Baseline characteristics of participants.

Parameters	Tuberculosis		
No (n = 16,385)	Yes (n = 72)	*p*-value
Age	57.95 ± 10.51	65.24 ± 10.7	<.0001
Age ≥ 65 year	4693 (28.64%)	44 (61.11%)	<.0001
Sex			0.2117
Male	7220 (44.06%)	37 (51.39%)	
Female	9165 (55.94%)	35 (48.61%)	
Smoking			0.2709
Nonsmoker	9952 (60.74%)	38 (52.78%)	
Ex-smoker	3551 (21.67%)	21 (29.17%)	
Current smoker	2882 (17.59%)	13 (18.06%)	
Alcohol consumption			0.8567
Non	4939 (30.14%)	20 (27.78%)	
Mild	10176 (62.11%)	47 (65.28%)	
Heavy	1270 (7.75%)	5 (6.94)	
Education ≥ high school	9454 (57.7%)	28 (38.89%)	0.0013
Household Income (Q1)	3048 (18.6%)	23 (31.94%)	0.0037
Physical Activity	6156 (37.57%)	26 (36.11%)	0.7986
Diabetes Mellitus	2196 (13.4%)	18 (25%)	0.004
Hypertension	6387 (38.98%)	32 (44.44%)	0.3429
Hypercholesterolemia	3546 (21.64%)	8 (11.11%)	0.0303
BMI	24.2 ± 2.92	23.37 ± 2.83	0.0158
Waist circumference	78.2 ± 9.17	78.19 ± 8.77	0.995
Glucose	101.73 ± 23.13	105.11 ± 33.74	0.217
SBP	122.08 ± 16.57	125.78 ± 16.23	0.0588
DBP	77.22 ± 10.15	75.49 ± 10.64	0.1491
Total Cholesterol	194.95 ± 36.31	183.69 ± 32.1	0.0087
HDL-C	49.5 ± 12.09	47.19 ± 10.16	0.1046
FEV_1_	92.39 ± 13.14	87.43 ± 16.72	0.0014
FVC	92.85 ± 11.6	88.67 ± 14.08	0.0023
FEV_1_/FVC	0.77 ± 0.07	0.74 ± 0.09	<.0001
AST*	21.88 (21.78-21.99)	22.98 (21.41-24.67)	0.1981
ALT*	19.29 (19.15-19.43)	19.43 (17.49-21.59)	0.8974
TG*	120.6 (119.55-121.67)	114.83 (101.26-130.21)	0.4695

Values are presented as number (%) or mean ± standard deviation. BMI: body mass index, SBP: systolic blood pressure, DBP: diastolic blood pressure, HDL-C: high density lipoprotein-Cholesterol, FVC: forced vital capacity, FEV1: forced expiratory volume in 1 second, AST: Aspartate Aminotransferase, ALT: Alanine Aminotransferase, TG: Triglyceride. * Geometric mean (95% C.I)

Values are presented as number (%) or mean ± standard deviation. BMI: body mass index, SBP: systolic blood pressure, DBP: diastolic blood pressure, HDL-C: high density lipoprotein – Cholesterol, FVC: forced vital capacity, FEV1: forced expiratory volume in 1 second, AST: Aspartate Aminotransferase, ALT: Alanine Aminotransferase, TG: Triglyceride. * Geometric mean (95% C.I)

There was no statistically significant difference in the prevalence of hypertension. BMI was significantly lower in participants with TB (23.37 ± 2.83 versus 24.2 ± 2.92, *p *= 0.0158). On the other hand, there was no meaningful difference in physical activity or waist circumference. Lung function was significantly lower in participants with TB than in those without TB (FEV_1_, 87.43 ± 16.72 versus 92.39 ± 13.14, *p *= 0.0014; FVC, 88.67 ± 14.08 versus 92.85 ± 11.6, *p *= 0.0023; FEV_1_/FVC, 0.74 ± 0.09 versus 0.77 ± 0.07, *p *= 0.0001).

### Incidence and risk of TB according to pattern of lung function impairment

Risk of incident TB by lung function status is shown in Table 3. Compared to individuals with normal lung function, risk of incident TB was higher in those with obstructive (HR: 2.033, 95% CI: 1.123–3.679) or restrictive lung function impairment (HR: 2.193, 95% CI: 1.120–4.294).

**Table 3:** Incidence and risk of tuberculosis according to pattern of lung function impairment.

Lung function pattern	Total no. (n)	TB cases (n)	TB incidence (per 1,000 person years)	HR (95% CI)		
Model 1	Model 2	Model 3
Normal	12666	37	0.53	1 (Ref.)	1 (Ref.)	1 (Ref.)
Obstructive pattern	2204	23	1.99	3.748 (2.227, 6.309)	2.070 (1.144, 3.744)	2.033 (1.123, 3.679)
Restrictive pattern	1587	12	1.42	2.681 (1.398, 5.141)	2.277 (1.168, 4.439)	2.193 (1.120, 4.294)

Model 1: non-adjusted; Model 2: adjusted for age, sex, BMI, smoking, alcohol consumption, household income and exercise; Model 3: adjusted for age, sex, BMI, smoking, alcohol consumption, household income, exercise, DM, hypertension and hypercholesterolemia

### Incidence and risk of TB according to degree of lung function impairment

Participants (n = 16,457) were divided into four quartile groups based on FEV_1_, FVC, and FEV_1_/FVC: lowest (Q1), lower middle (Q2), higher middle (Q3), and highest (Q4) (Table 4). Based on FEV1, incidence of TB was higher in the lowest group (Q1) than in the highest group (Q4) (HR: 1.914, 95% CI: 1.047–3.501). Higher incident TB was observed with decreasing quartiles of FVC and FEV1/FVC, although no significant association was found.

**Table 4:** Incidence and risk of tuberculosis according to degree of lung function impairment (quartile).

Lung Function	Total no. (n)	TB cases (n)	TB incidence (per 1,000 person years)	HR (95% CI)		
Model 1	Model 2	Model 3
FEV1						
Q4 (highest)	4114	17	0.75	1 (Ref.)	1 (Ref.)	1 (Ref.)
Q3	4114	11	0.49	0.652 (0.305, 1.392)	0.783 (0.365, 1.681)	0.776 (0.362, 1.666)
Q2	4114	12	0.53	0.711 (0.339, 1.488)	0.855 (0.405, 1.805)	0.854 (0.404, 1.803)
Q1 (lowest)	4114	32	1.47	1.962 (1.090, 3.534)	1.941 (1.063, 3.544)	1.914 (1.047, 3.501)
FVC						
Q4	4114	14	0.61	1(Ref.)	1(Ref.)	1(Ref.)
Q3	4114	14	0.62	1.002 (0.478, 2.102)	1.066 (0.507, 2.24)	1.07 (0.509, 2.250)
Q2	4115	18	0.80	1.308 (0.651, 2.630)	1.331 (0.658, 2.692)	1.348 (0.666, 2.726)
Q1	4114	26	1.19	1.944 (1.015, 3.724)	1.660 (0.850, 3.239)	1.64 (0.838, 3.213)
FEV_1_/FVC						
Q4	4114	12	0.53	1(Ref.)	1(Ref.)	1(Ref.)
Q3	4114	8	0.35	0.669 (0.273, 1.636)	0.524 (0.213, 1.291)	0.526 (0.214, 1.296)
Q2	4115	21	0.94	1.785 (0.878, 3.628)	1.114 (0.534, 2.327)	1.127 (0.539, 2.357)
Q1	4114	31	1.42	2.692 (1.382, 5.243)	1.169 (0.544, 2.509)	1.177 (0.548, 2.529)

Model 1: non-adjusted; Model 2: adjusted for age, sex, BMI, smoking, alcohol consumption, household income and exercise; Model 3: adjusted for age, sex, BMI, smoking, alcohol consumption, household income, exercise, DM, hypertension and hypercholesterolemia

**Fig 2:**
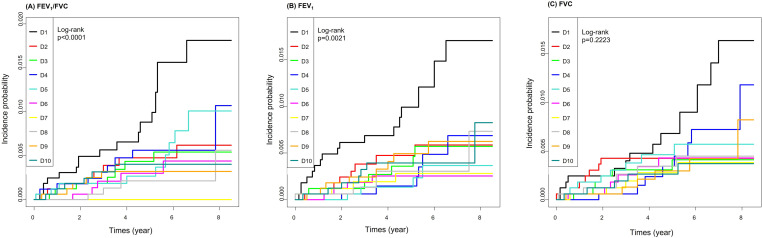
Cumulative incidence curves of tuberculosis according to lung function decile by Kaplan-Meier method and log-rank test.

Fig 2 shows cumulative incidence probability of TB according to lung function decile. The incidence probability of TB was significantly higher in lower deciles of FEV1/FVC and FEV1 (log-rank *p* < 0.0001 for FEV1/FVC, *p* = 0.0021 for FEV1). Table 5 shows incidence and risk of TB according to lung function decile. In FEV1 deciles, the lowest group (D1) had an increased risk of TB compared to the highest group (D10) in model 3 (adjusted HR: 2.762, 95% CI: 1.139–6.696). In FVC and FEV1/FVC deciles, TB risk was not significantly different between the lowest decile and other deciles in model 3.

**Table 5:** Incidence and risk of tuberculosis according to degree of lung function impairment (decile).

Lung Function	Total no. (n)	TB cases (n)	TB incidence (per 1,000 person years)	HR (95% CI)		
Model 1	Model 2	Model 3
FEV1						
D10 (highest)	1645	7	0.77	1(Ref.)	1(Ref.)	1(Ref.)
D9	1646	8	0.87	1.125 (0.408, 3.101)	1.431 (0.516, 3.970)	1.432 (0.516, 3.976)
D8	1646	5	0.56	0.717 (0.228, 2.259)	1.009 (0.317, 3.212)	1.017 (0.320, 3.240)
D7	1646	4	0.57	0.574 (0.168, 1.960)	0.783 (0.227, 2.694)	0.788 (0.229, 2.714)
D6	1645	4	0.44	0.568 (0.166, 1.942)	0.792 (0.229, 2.739)	0.78 (0.226, 2.697)
D5	1646	4	0.44	0.569 (0.167, 1.945)	0.817 (0.236, 2.822)	0.806 (0.233, 2.786)
D4	1646	6	0.66	0.851 (0.286, 2.534)	1.178 (0.391, 3.550)	1.197 (0.397, 3.609)
D3	1646	7	0.79	1.019 (0.357, 2.906)	1.372 (0.473, 3.979)	1.385 (0.477, 4.023)
D2	1646	8	0.93	1.198 (0.434, 3.304)	1.514 (0.541, 4.237)	1.485 (0.530, 4.164)
D1 (lowest)	1645	19	2.17	2.801 (1.178, 6.664)	2.778 (1.149, 6.716)	2.762 (1.139, 6.696)
FVC						
D10	1645	5	0.56	1(Ref.)	1(Ref.)	1(Ref.)
D9	1646	6	0.65	1.164 (0.355, 3.813)	1.25 (0.381, 4.102)	1.204 (0.367, 3.956)
D8	1646	6	0.65	1.178 (0.359, 3.859)	1.285 (0.391, 4.217)	1.255 (0.382, 4.124)
D7	1646	5	0.55	0.994 (0.288, 3.434)	1.092 (0.315, 3.784)	1.060 (0.306, 3.676)
D6	1646	6	0.66	1.190 (0.363, 3.899)	1.278 (0.389, 4.205)	1.271 (0.386, 4.183)
D5	1645	8	0.89	1.604 (0.525, 4.902)	1.706 (0.555, 5.245)	1.675 (0.544, 5.155)
D4	1646	8	0.89	1.602 (0.524, 4.897)	1.687 (0.548, 5.188)	1.669 (0.542, 5.139)
D3	1646	6	0.67	1.208 (0.369, 3.959)	1.224 (0.371, 4.041)	1.229 (0.371, 4.069)
D2	1646	7	0.81	1.458 (0.463, 4.593)	1.382 (0.433, 4.414)	1.345 (0.420, 4.306)
D1	1645	15	1.73	3.118 (1.133, 8.580)	2.488 (0.885, 6.991)	2.370 (0.838, 6.700)
FEV1/FVC						
D10	1645	6	0.65	1(Ref.)	1(Ref.)	1(Ref.)
D9	1614	5	0.56	0.853 (0.260, 2.796)	0.784 (0.239, 2.573)	0.783 (0.239, 2.572)
D8	1678	4	0.43	0.651 (0.184, 2.308)	0.523 (0.147, 1.861)	0.516 (0.145, 1.839)
D7	1675	0	0	0	0	0
D6	1611	5	0.57	0.868 (0.265, 2.844)	0.617 (0.186, 2.043)	0.630 (0.190, 2.090)
D5	1638	9	1.00	1.528 (0.544, 4.293)	0.970 (0.338, 2.784)	0.984 (0.342, 2.833)
D4	1659	9	1.01	1.541 (0.548, 4.329)	0.888 (0.307, 2.567)	0.894 (0.309, 2.592)
D3	1648	7	0.79	1.202 (0.404, 3.578)	0.632 (0.204, 1.959)	0.648 (0.209, 2.012)
D2	1649	8	0.91	1.383 (0.480, 3.987)	0.621 (0.202, 1.909)	0.619 (0.201, 1.909)
D1	1640	19	2.22	3.387 (1.352, 8.483)	1.178 (0.417, 3.328)	1.183 (0.417, 3.350)

Model 1: non-adjusted; Model 2: adjusted for age, sex, BMI, smoking, alcohol consumption, household income and exercise; Model 3: adjusted for age, sex, BMI, smoking, alcohol consumption, household income, exercise, DM, hypertension and hypercholesterolemia

## Discussion

In most previous studies regarding the relationship between pulmonary function and pulmonary TB, it has been shown that patients who have suffered from pulmonary TB have a decrease in pulmonary function over time [3–6,17]. Unlike previous research studies, however, we investigated how baseline lung function, not the change in lung function after pulmonary TB, affected the development of TB. In our study, baseline lung function of participants who developed TB was significantly lower than that of participants without TB. The risk of TB was higher in participants with obstructive or restrictive lung function impairment than in participants with normal lung function. Furthermore, TB incidence was inversely correlated with FEV_1_.

There are several factors causing lung function decline. Smoking is well known as a factor that can reduce lung function. Many studies have already demonstrated the relationship between smoking and lung function decline [18–21]. Cigarette smoke contains harmful chemicals and particles that can directly damage lungs and airways, leading to inflammation and scarring that can cause airflow limitation. Smoking also causes oxidative stress in lungs, which can damage cells and tissues and lead to inflammation and reduced lung function. Over time, this damage can lead to the development of chronic lung diseases such as chronic obstructive pulmonary disease (COPD), which can cause serious lung function decline and severely impact quality of life [22,23]. SES is also associated with decreased lung function [24]. In a population-based cohort analysis, Jackson *et al*. have reported that the lower the SES in childhood, the earlier and faster the decline in lung function [25]. A longitudinal study has shown that socioeconomic position across the life course could have a significant impact on lung function decline in later life [26]. In other words, it has been known that there is an inverse association between SES and lung function, with lower SES being associated with poorer lung function. Several factors might contribute to this relationship. One potential reason is that people with lower SES are more likely to live in areas with poor air quality, which can lead to respiratory problems and decreased lung function [27]. Additionally, people with lower SES might have less access to healthcare and might be less likely to receive preventive care, which can also contribute to decreased lung function [28]. Other factors such as stress, lack of physical activity, poor diet, and exposure to environmental biomass may also play a role in lung function decline among people with lower SES [29]. One study has followed a cohort of individuals over time and found that long-term exposure to household air pollution from solid fuel use is associated with declining lung function [30]. Overall, the relationship between lung function and SES is complex and multifactorial. Low SES is associated with several factors that can negatively impact lung health, making individuals with low SES more vulnerable to poor lung function.

What is noteworthy here is that smoking, low SES, and environmental biomass exposure mentioned above are related not only to the deterioration of lung function, but also to the occurrence of pulmonary TB. One study has shown that smokers are more likely to develop TB than non-smokers and that the risk increases with increasing number of cigarettes smoked per day [31]. A systematic review of observational studies has found that smokers have a 1.5 to 2 times higher risk of TB than non-smokers [32]. Smoking has been found to negatively impact the immune system, making it more difficult for the body to fight off TB. It can also impair the effectiveness of TB treatment, increasing the risk of treatment failure and drug-resistant TB [33]. In addition, there are several studies that support the relationship between low SES and an increased risk of TB [34–36]. Those studies have shown that individuals with lower income, those with lower education levels, and those living in poverty are more likely to contract TB. This is due to a variety of factors, such as overcrowded living conditions, poor nutrition, and limited access to healthcare, which can weaken the immune system and make individuals more vulnerable to the disease. Environmental biomass exposure has also been associated with the development of TB. Several studies have shown that prolonged exposure to indoor air pollution from biomass fuels can have a negative impact on respiratory health and increase the risk of TB [37–39]. This is due to the damaging effects of smoke on the lungs and immune system, which can make individuals more susceptible to the disease. Previous studies have also shown that exposure to biomass fuel smoke is associated with increased TB transmission, as infected individuals who are exposed to the smoke might be more likely to spread the disease to others.

As mentioned above, smoking, low SES, and exposure to environmental pollutants can affect lung function and the occurrence of tuberculosis. Therefore, we analyzed the relationship between lung function and the occurrence of tuberculosis through multivariate analysis after adjusting for several factors (smoking, BMI, alcohol consumption, household income, and regular physical activity) related this. Considering that smoking, low SES, and exposure to environmental pollutants, which can cause deterioration of lung function, are all related to the occurrence of TB, lung function decline might also be related to the development of TB. However, this relationship may not be a direct cause-and-effect one, but rather mediated by factors such as smoking, lower SES, and environmental pollutants exposure. Further research is needed to better understand this relationship.

It is well-known that TB caused by *Mycobacterium tuberculosis* is an infectious disease that primarily affects the lungs. TB bacteria can spread from person to person through the air when an infected person talks, coughs, or sneezes [40–42]. The lungs are responsible for filtering out harmful substances, including bacteria, from the air we breathe [43,44]. When lung function is impaired, the ability to filter out these substances is reduced, making it easier for TB bacteria to enter the body and cause an infection. In addition, decreased lung function causes inflammation of the airways [45,46], which can lead to mucociliary dysfunction [47]. In cases of mucociliary dysfunction, the cilia are unable to move effectively and the mucus becomes stagnant, creating an environment that is conducive to bacterial growth. This can increase the risk of infection, including TB [48]. Meanwhile, some studies have reported that decreased lung function is related to systemic inflammation or macrophage activation [49–51], which can increase susceptibility to TB [52–57]. Systemic inflammation is associated with an increased release of pro-inflammatory cytokines such as tumor necrosis factor (TNF) and interleukin (IL)-1. High levels of these pro-inflammatory cytokines can suppress the immune response, making it more difficult for the body to control the TB infection. Additionally, elevated levels of pro-inflammatory cytokines can create a favorable environment for the growth and spread of TB bacterium [54–57]. Macrophage activation is a critical aspect of the host’s immune response to *Mycobacterium tuberculosis*. However, macrophage activation can also increase susceptibility to pulmonary TB infection in some cases [52,53]. Activated macrophages can increase the production of pro-inflammatory cytokines and suppress the immune response as described above, thereby leading to a suppressed body’s defense against to TB infection. In addition, activated macrophages can promote the formation of granulomas, which are clusters of immune cells that form in response to *Mycobacterium tuberculosis*. While granulomas can initially limit the spread of *Mycobacterium tuberculosis*, they can also create a microenvironment that favors the persistence of TB bacterium [58,59]. This is because oxygen levels within granulomas can become low, creating conditions that are unfavorable for many bacteria-killing immune cells, but ideal for *Mycobacterium tuberculosis*. Furthermore, activated macrophages can cause tissue damage and remodeling, which can create more favorable conditions for *Mycobacterium tuberculosis* to persist and multiply. These processes can also contribute to the formation of TB cavities, which are large air spaces within the lung tissue that are filled with *Mycobacterium tuberculosis* and other infectious agents [52,53]. However, the extent to which these factors contribute to the incidence of TB can vary depending on the individual and other factors.

Several studies have reported results similar to ours [60–62]. Jick *et al*. have conducted a case-control study and reported that the use of glucocorticoids is a risk factor for TB [61]. They additionally identified obstructive pulmonary disease as an independent risk factor for TB [61]. However, their evaluation of obstructive pulmonary disease was based solely on diagnosis using medical records without directly analyzing lung function. Benfield *et al*. have reported that the risk of TB is increased as lung function decreases [60]. However, since their study only analyzed patients with pulmonary TB who required hospitalization, it was difficult to conclude that they evaluated the total incidence of TB. Furthermore, while they reported that the incidence of pulmonary TB increased as the COPD GOLD stage decreased compared to normal lung function, our study demonstrated an increased risk of TB even with a decrease in FEV1 alone regardless of the FEV1/FVC ratio. Inghammar *et al*. have also reported that the risk of TB increases as lung function declines [62]. However, their study had a small number of TB cases without providing data on past TB cases or adjusting for other risk factors for TB such as comorbidity or corticosteroid use.

Our study also has some limitations. We could not assess the previous history of respiratory conditions that could potentially affect lung function due to data limitation. We could not entirely rule out the effect of confounding factors such as use of corticosteroids or other immunosuppressants or latent TB infection, which could lead to increased susceptibility to TB infection. We could not find out whether active TB infection was reactivated from latent phase or newly infected either. In the multivariate analysis (Table 5), the lowest decile (D1 for FEV1) showed a wide range of confidence intervals in all the three models (Model 1, 2, and 3). This is because the incidence of TB itself is low in healthy adults, and therefore the case number of TB is small. Although our study has these limitations, it deserves attention that it was able to suggest the impaired lung function itself could be a risk factor for TB in a nationwide large-scale study targeting the general population. To address these limitations, our study should be supplemented by additional, well-designed, larger-scale follow-up studies.

## Conclusions

In conclusion, participants with lower lung function had an increased risk of TB than those with normal lung function in a nationwide population-based cohort study. We also identified that TB incidence was inversely correlated with FEV_1_. Additional large-scale prospective study is required to clarify the mechanism of the relationship between poor lung function and increased risk of TB. Furthermore, if positive data regarding this are accumulated, our study could serve as a basis for judging people with impaired lung function as a risk group that requires targeted prevention against TB.
